# Feasibility and Reliability of Two-Dimensional Shear-Wave Elastography of the Liver of Clinically Healthy Cats

**DOI:** 10.3389/fvets.2020.614750

**Published:** 2020-12-23

**Authors:** Kyeonga Kim, Jieun Lee, Jaebeom So, Yong-seok Jang, Mingyu Jung, Kyuyong Kang, Mincheol Choi, Junghee Yoon

**Affiliations:** College of Veterinary Medicine and the Research Institute for Veterinary Science, Seoul National University, Seoul, South Korea

**Keywords:** elastography, feline, free breathing, liver, shear-wave elastography, ultrasonography

## Abstract

Given the broad overlap of normal and abnormal liver tissue in the subjective evaluation of the liver in conventional B-mode ultrasonography, there is a need for a non-invasive and quantitative method for the diagnosis of liver disease. Novel two-dimensional shear-wave elastography (2-D SWE) can measure tissue stiffness by propagation of the shear wave induced using acoustic radiation force impulse in real time. To the best of our knowledge, two-dimensional shear-wave measurement of the liver in cats has not been reported to date. This study assessed the feasibility, reliability, normal values, and related influencing factors of 2-D SWE for assessment of the feline liver without anesthesia and breath-holding. Two-dimensional shear-wave ultrasonography was performed by two evaluators at the right and left sides of the liver. Twenty-nine client-owned clinically healthy adult cats were included. The means and standard deviations for the shear-wave speed and stiffness in the right liver were 1.52 ± 0.13 m/s and 6.94 ± 1.26 kPa, respectively, and those for the left liver were 1.61 ± 0.15 m/s and 7.90 ± 1.47 kPa, respectively. Shear-wave speed (*P* = 0.005) and stiffness (*P* = 0.002) were significantly lower in the right liver when compared to the left. The intraclass correlation value for liver stiffness was 0.835 and 0.901 for the right and left liver, respectively, indicating high interobserver agreement. Age, weight, body condition score (BCS), gabapentin administration, and measurement depths were not significantly correlated with liver stiffness or elastography measurements (*P* > 0.05). Our findings suggest that 2-D SWE measurements of the liver are not influenced significantly by age, weight, or BCS and can be reliably performed without anesthesia and breath-holding in cats. The values determined here can help form the basis for reference elastography values for evaluation of the feline liver.

## Introduction

Feline liver diseases include cholangiohepatitis, chronic hepatitis, hepatic lipidosis, and hepatic neoplasia. Inflammatory liver disease is reported to be the most common, or second most common, abnormality detected in feline liver biopsies. Inflammatory liver disease accounted for 50% of abnormal feline liver biopsy samples in Japan (1) and 25.7% of those in the United States. Hepatic lipidosis accounted for 50% of the abnormal feline liver biopsy samples in the United States (2). The prevalence of hepatic lipidosis was reported as 0.16% in a large feline population of primary care practices in the United States (3). Most cats with inflammatory liver disease recover with appropriate treatment. The recovery rate of hepatic lipidosis is 80% in the absence of fatal underlying conditions with appropriate nutritional support. However, prognosis could be affected by the time of initiating the proper treatment or concurrent disease (4, 5). Therefore, a quick and accurate diagnosis would be important to properly treat these diseases. Liver diseases in small animal veterinary medicine are often evaluated by ultrasonography. However, there is a broad overlap of normal and abnormal liver tissue in the ultrasonographic evaluation. Traditional ultrasonography assessment of diffuse hepatic disease is typically subjective, and abnormalities cannot be differentiated, particularly when changes are mild or in the early course of a disease (6).

Given the limitation of traditional ultrasonography, the definitive diagnosis of most liver disease depends on the histopathological examination of the liver tissue (1). Despite liver biopsy being the gold standard for diagnosing liver disease, it is invasive and may require anesthesia due to the lack of cooperation by the patient. There is a need for a non-invasive, safe method with high diagnostic accuracy (7, 8). As one of these methods, elastography involves a non-invasive evaluation of the mechanical characteristics of tissue by means of ultrasonic imaging and mostly used to evaluate diffuse liver disease in human medicine, such as chronic hepatitis, non-alcoholic fatty liver disease, alcoholic liver disease, and portal hypertension. Elastography has recently become widely available on recent commercial ultrasound machines. Tissue stiffness can be measured using various methods. In strain elastography, strain is induced by manual compression or pulsation. In transient elastography (TE), mechanical vibrations generate shear waves. Shear-wave speed can also be created by acoustic radiation force impulse (ARFI) using ultrasound beams, and assessment can be made either at one point, as in point shear-wave elastography (pSWE), or by using several ARFI lines, as in two-dimensional shear-wave elastography (2-D SWE) (9).

The novel 2-D SWE technique can measure propagation of the shear wave induced by ARFI in real time. Shear-wave propagation visualization and a color-coded map of elasticity are superimposed on the B-mode ultrasonography image of the liver parenchyma, which is helpful for anatomic and tissue stiffness guidance. The region of interest (ROI) can be adjusted for size and location to assess a targeted measurement area (10). Furthermore, 2-D SWE can be performed with the subject breathing freely, as conducted in the human pediatric population (11). Considering the difficulty in obtaining cooperation from veterinary patients, 2-D SWE may be a very useful method.

Various recent veterinary investigations have shown that in dogs, 2-D SWE can be applied for the estimation of stiffness in normal parenchyma of the liver, spleen, kidneys, pancreas, prostate, lymph nodes, submandibular salivary gland, thyroid (12, 13), postpartum uterine wall (14), small intestinal wall (15), and thigh muscle (16). Moreover, several studies attempted to compare abnormal lesions of the liver, pancreas, and kidneys with those of normal animals. 2-D SWE successfully differentiated artificially ablated lesions of the liver (17) and spontaneous hepatic fibrosis of the liver (18) from the normal parenchyma in dogs, with a significant increase in stiffness value discovered. The shear-wave velocity was increased in dogs with pancreatic disease (19). The stiffness of the renal cortex and medulla was significantly higher in cats with chronic kidney disease than in normal cats (20).

Given the future potential utilization of 2-D SWE as a non-invasive, quantitative method in small animal clinics, as in human medicine, further research on elastography would be valuable, particularly in terms of the liver parenchyma, where elastography is most often utilized in human medicine. Nevertheless, 2-D SWE measurement of the liver in cats has not been reported to date. To utilize 2-D SWE for evaluation of feline liver disease, studies on both healthy and diseased subjects are necessary. In the present study, we hypothesized that 2-D SWE measurements could be conducted without anesthesia and breath-holding in feline patients, as in human pediatric subjects (11). This study therefore aimed to assess the feasibility and reliability of, and the factors influencing, 2-D SWE of the feline liver without anesthesia and breath-holding. Normal values of shear-wave speed and stiffness in the clinically normal adult feline liver were determined.

## Materials and Methods

### Animals

This study was conducted under the approval of the Seoul National University Institutional Animal Care and Use Committee (SNU-IACUC approval number: SNU-200301-1). Thirty-five client-owned clinically healthy adult cats without history or clinical signs of liver or systemic diseases were enrolled. If requested by the owner, gabapentin (100 mg/cat) was prescribed to reduce stress during transport and examination. Routine physical examination; blood tests, including complete blood count; and serum biochemistry, including alanine aminotransferase (ALT), aspartate aminotransferase (AST), alkaline phosphatase (ALP), gamma-glutamyl transferase (GGT), total bilirubin, albumin, blood urea nitrogen (BUN), glucose, and cholesterol concentrations, were performed in all cats.

We evaluated 35 cats using 2-D SWE. Of these, six cats were excluded due to unreliable 2-D SWE measurements [three cats were excluded because the propagation lines in propagation maps had a lack of parallel appearances; technical failure. The other three were excluded because the interquartile range (IQR)/median exceeded 0.3; unreliable measurements]. Twenty-nine clinically healthy cats were included in this process of establishing reference values for 2-D SWE of the feline liver.

### Ultrasonographic Examination

All cats fasted for 8 h. Routine ultrasonography was performed with an ultrasonography scanner (Aplio i800, Canon Medical Systems, Tochigi, Japan) after clipping the subject's hair and applying coupling gel. Conventional B-mode ultrasonography was performed with a 7.0-MHz convex transducer (PVT-712BT, Canon Medical Systems, Tochigi, Japan) and a 12.0-MHz linear transducer (PLI-1205BX, Canon Medical Systems, Tochigi, Japan) before 2-D SWE measurement. Real-time morphologic evaluation of the liver parenchyma and vascular and biliary structures was performed.

After conventional B-mode ultrasonography, 2-D SWE measurements were performed with a 12.0-MHz linear transducer. SWE measurements were obtained by two veterinarians with diagnostic imaging expertise (KAK and JL) following the guidelines and recommendations for liver ultrasonography elastography in human medicine (21) and previous studies of liver elastography in dogs (12). Measurements were performed on the right and left sides of the liver, with the cats positioned in lateral recumbency. The right liver was defined as the right portion of the liver located on the right side of the gallbladder and cranial to the right kidney. The left liver was defined as the left portion of the liver, located on the left side of the gallbladder. The probe was positioned perpendicular to the body wall in the intercostal space, without pressure, to visualize the liver at the location of the best acoustical window. 2-D SWE measurement was performed at the end-expiratory phase to minimize the effect of breathing motion. Shear-wave speed was measured and converted to elastic modulus as Young's modulus, derived by using the equation $E=3\rho {c_{s}}^{2}$, where *E* = Young's modulus, *c*_*s*_ = shear-wave speed, and ρ = tissue density (22).

**Figure 1:**
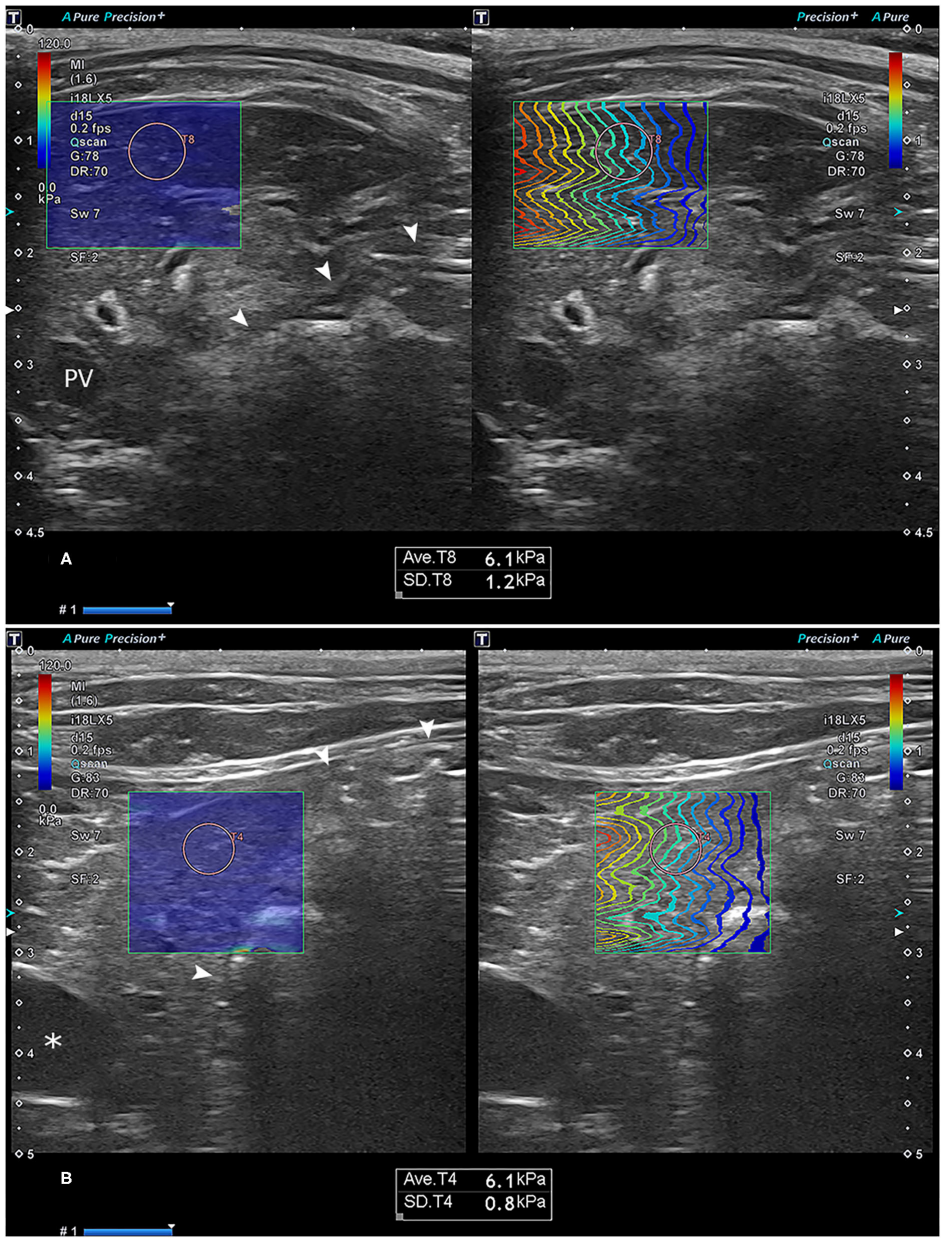
Representative images of the two-dimensional shear-wave elastography measurement in the right **(A)** and left **(B)** liver. Note the optimal color-fill in the stiffness color mapping (left) and parallel wave propagation of the propagation map (right) in each site of the liver. The region-of-interest (ROI) circle, set to a 5-mm size, was placed in the liver parenchyma by avoiding the vessels and bile ducts. The text box below the ultrasonographic image shows the mean and standard deviation of the stiffness in the ROI. Asterisk: gallbladder; arrowhead: stomach. PV, portal vein.

In addition to the B-mode image, color mapping of the shear-wave speed (m/s) and a propagation mapping were visualized. The ROI diameter was set to 5 mm, with an ROI depth of up to 4.5 cm. When the proper B-mode image, color map, and propagation map of the hepatic parenchyma were acquired, 2-D SWE measurement was performed to avoid vessels and bile ducts (Figure 1). 2-D SWE measurements were expressed as shear-wave speed (m/s) and liver stiffness (kPa). Median values of the shear-wave speed and liver stiffness were obtained by five measurements for each side of each cat. Median values of the measurement depth were recorded.

**Figure 2:**
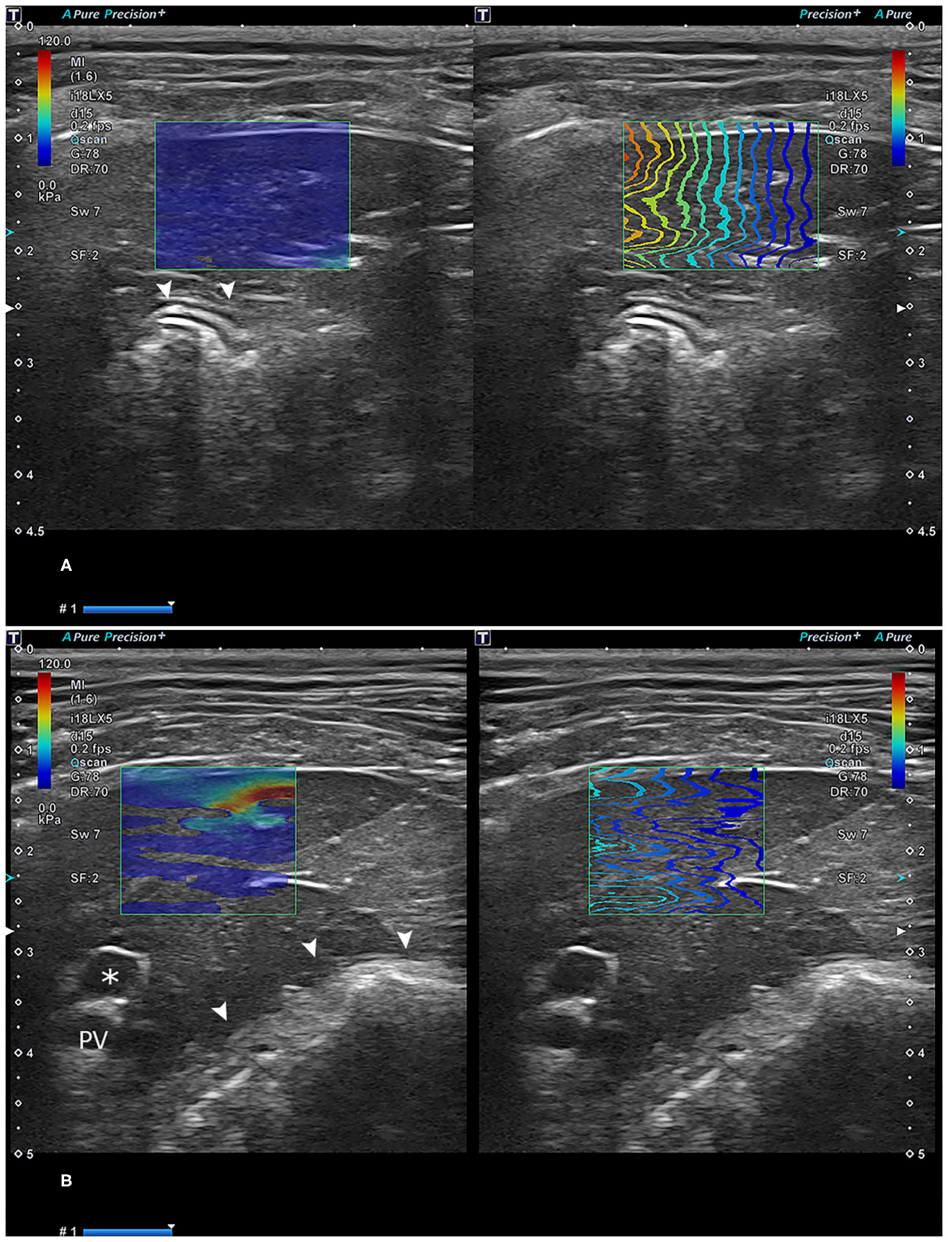
Reliable **(A)** and unreliable **(B)** two-dimensional shear-wave elastography images. In **(A)**, the propagation lines are linear and parallel to each other. In **(B)**, lines are not linear, with some unconnected parts with a lack of parallel appearance. Asterisk: gallbladder; arrowhead: stomach. PV, portal vein.

The propagation direction of the shear wave was indicated by the color of the contour lines. Measurements with optimal color-fill in the stiffness color mapping and parallel wave propagation in propagation maps were considered reliable (Figure 2). A stiffness value <1 kPa was considered an invalid measurement. In addition, the IQR divided by median (IQR/median) had to be <0.3 for kPa measurements and <0.15 for m/s measurements (9). The inability to obtain an adequate color mapping and propagation maps was considered a technical failure, and IQR/median values of more than 0.3 for kPa measurements or more than 0.15 m/s were considered unreliable (23).

### Statistical Analysis

Statistical analyses were performed using SPSS software (version 25; IBM Corporation, Armonk, NY, USA). The mean and standard deviation values of the right and left liver were obtained. Interobserver differences between the two observers for each measurement were evaluated using the intraclass correlation coefficient (ICC). The normality of data distribution for all variables was assessed with the Shapiro–Wilk test for normality. Shear-wave speed (m/s) and liver stiffness (kPa) between the left and right sides of the liver were compared using a paired *t*-test. To analyze the effect of age, weight, body condition score (BCS), depth of the measurement, and gabapentin administration on right liver stiffness (kPa), Spearman's rank correlation coefficient or the Mann–Whitney *U* test was used, as appropriate. A multivariate logistic regression model was used to assess factors associated with unreliable measurements including age, weight, BCS, and gabapentin administration.

## Results

### Animals

We evaluated 35 cats using 2-D SWE, and data of six cats were excluded due to unreliable 2-D SWE measurements. Thus, data from 29 cats were included in the study. These cats included three intact females, 10 neutered females, and 16 neutered males and comprised the following breeds: 16 domestic shorthairs, three Ragdolls, two Siameses, and one each of Devon Rex, Norwegian Forest, American Shorthair, British Shorthair, Persian, Russian Blue, Bengal, and mixed breed cat. The mean and standard deviation of the age of the cats were 3.37 ± 1.06 years (range: 1–7 years). The mean and standard deviation of the cats' body weight were 4.86 ± 1.52 kg (range: 2.42–8.94 kg). The mean and standard deviation of the cats' BCS were 5.41 ± 1.45 (range: 3–8). The mean and standard deviation of the depth of the measurement were 1.61 ± 0.38 cm (range: 1.00–2.30 cm). Cats with a BCS ≥ 6 were considered overweight (24).

### Liver Shear-Wave Measurements

**Figure 3:**
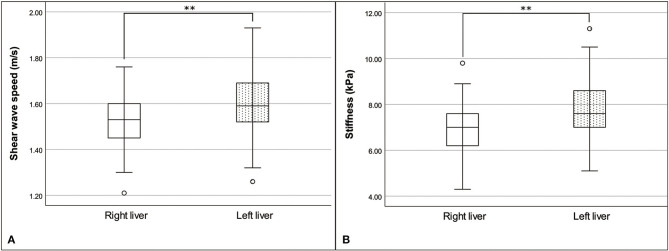
Box and whisker plot of the median shear-wave speed **(A)** and stiffness **(B)** of the liver, with a comparison between the right and left liver. Shear-wave speed and stiffness were significantly different between the liver, by paired *t*-test; **P < 0.01.

The means and standard deviations for the median shear-wave speed and stiffness in the right liver were 1.52 ± 0.13 m/s (range: 1.21–1.76 m/s) and 6.94 ± 1.26 kPa (range: 4.30–9.80 kPa), respectively. The means and standard deviations for the median shear-wave speed and stiffness in the left liver were 1.61 ± 0.15 m/s (range: 1.26–1.93 m/s) and 7.90 ± 1.47 kPa (range: 5.10–11.30 kPa), respectively. Median shear-wave speed (*P* = 0.005) and stiffness (*P* = 0.002) in the right liver were significantly lower than those in the left liver (Figure 3). The ICC value of the liver stiffness was 0.835 for the right liver and 0.901 for the left liver, indicating high interobserver agreement.

### Associated Factors

According to the Spearman's rank correlation coefficient, age, weight, and depth of the measurement were not significantly correlated with liver stiffness (age: *r* = −0.053, *P* > 0.05; weight: *r* = 0.110, *P* > 0.05; depth: *r* = 0.245, *P* > 0.05). According to the Mann–Whitney *U*-test, BCS ≥ 6 (13/29 cats) and gabapentin administration (9/29 cats) were not significantly correlated with liver stiffness (*P* > 0.05). The mean and standard deviation for liver stiffness in cats with BCS ≥ 6 were 7.06 ± 1.37 kPa (range: 4.90–8.90 kPa) and for those with BCS < 6 were 6.85 ± 1.19 kPa (range: 4.30–9.80 kPa). The mean and standard deviation for the liver stiffness were 6.93 ± 1.17 kPa (range: 4.90–8.60 kPa) and 6.95 ± 1.32 kPa (range: 4.30–9.80 kPa) with and without gabapentin administration, respectively.

A total of 35 cats, including six with technical failure and unreliable measurements, were analyzed for the correlation of patient factors. The number of cats with BCS ≥ 6 (overweight) was 18/35. The number of cats in whom gabapentin was administered was 12/35. Technical failure and unreliable measurements did not have significant correlation with age, weight, BCS, and gabapentin administration (*P* > 0.05). The percentage of failure and unreliable 2-D SWE measurements in overweight patients was 27.78% (5/18), and in non-overweight patients, it was 5.88% (1/17). The percentage of failure and unreliable 2-D SWE measurements in cats in whom gabapentin was administered was 25% (3/12) and that without gabapentin administration was 13.04% (3/23).

## Discussion

In most cases, sedation or anesthesia is essential to perform somewhat aggressive or invasive procedures in veterinary clinical routines. It would be best practice if essential care procedures could be performed without such procedures, ensuring safety while providing critical diagnostic clues in patient care. Two-dimensional SWE can be conducted in free-breathing status in human pediatric medicine (11). This is considered relevant and applicable without anesthesia in veterinary patients.

To the best of our knowledge, no previous study has reported 2-D SWE measurements in the feline liver. In this study, 2-D SWE measurements of feline livers were performed under circumstances similar to those for routine clinical situations without anesthesia. The shear-wave speed and stiffness in the right liver were 1.52 ± 0.13 m/s and 6.94 ± 1.26 kPa, respectively, and those in the left liver were 1.61 ± 0.15 m/s and 7.90 ± 1.47 kPa, respectively. The 2-D SWE was measured in healthy dogs in two previous studies. In one of those, liver stiffness values measured using the intercostal approach were 7.57 ± 1.17 kPa (5–10-mm depth) and 8.36 ± 1.16 kPa (10–15-mm depth) in the right liver and 7.8 ± 0.06 kPa (5–10-mm depth) and 8.1 ± 0.38 kPa (10–15-mm depth) in the left liver (13). In the other study, liver stiffness values were 6.93 ± 0.79 kPa in the right liver and 6.02 ± 0.61 kPa in the left liver (12). These values are similar to those of our study on feline livers. The shear-wave speed and stiffness in feline livers evaluated in our study can contribute to establishing reference elastography values for healthy feline livers.

2-D SWE measurements were attempted in 35 cats and were successful in 29, yielding an 82.9% success rate. Measurements in six cats were considered as technical failure and unreliable measurements and, thus, excluded. Three cats were excluded because the propagation lines in propagation maps had a lack of parallel appearances: technical failure. The other three were excluded for IQR/medians exceeding 0.3: unreliable measurements. The proportions of technical failure and unreliable measurements were 8.6% in each case. In the systematic review of 2-D SWE in humans, the proportion of technical failure was 2.3%, and the proportion of unreliable measurements was 7.5% (23). Considering that the studies in human medicine are conducted under breath-holding condition and with the full cooperation of the patient, this success rate can be accepted as favorable. ICCs were 0.835 for the right liver and 0.901 in the left liver, indicating high interobserver agreement. These results suggest the clinical applicability of elastography in cats. Reliable results could be obtained without anesthesia or breath-holding due to the rapid measurement process (11) and availability of real-time evaluation of the propagation map and B-mode ultrasonography image of the liver parenchyma to choose an appropriate ROI in 2-D SWE.

In human medicine, elastography is mostly used to evaluate diffuse liver disease, and elastography techniques are widely implemented. Abnormalities in organs other than the liver, such as the breast, thyroid, gastrointestinal tract, prostate, and musculoskeletal system, can be evaluated using strain and shear-wave elastography as appropriate (25, 26). Chronic liver damage results in hepatic fibrosis characterized by an increase of extracellular matrix produced by fibroblast-like cells. Elastography can be used in staging the degree of fibrosis in chronic hepatitis by measuring stiffness (22). In veterinary medicine, a recent study showed a significant difference in the 2-D SWE between dogs with clinically relevant hepatic fibrosis and those without clinically relevant hepatic fibrosis (18). Inflammatory liver disease and hepatic lipidosis are the most common hepatobiliary disorders in feline patients. There are three forms of feline inflammatory liver disease: neutrophilic, lymphocytic, and chronic cholangitis. In these diseases, inflammatory cells such as neutrophils or lymphocytes infiltrate the portal areas and hepatic parenchyma. Biliary hyperplasia occurs, and there are various degrees of periportal and periductal fibrosis. These infiltrative and fibrotic changes can induce an increase in stiffness of the liver, just like in human patients with chronic hepatitis (27). In non-alcoholic fatty liver disease of humans, shear-wave velocity was lower in patients with simple steatosis than in healthy volunteers. It was hypothesized that since there is fat deposition in the liver parenchyma, the liver becomes softer (28). In feline hepatic lipidosis, lipid accumulation occurs within the hepatocytes, similar to the non-alcoholic fatty liver disease of humans (29). It can be expected that feline hepatic lipidosis would induce the lower shear-wave velocity in cats.

2-D SWE values in the right and left liver differed significantly in the present study. Similarly, there was a significant difference in the shear-wave velocity between the right and left liver in the 2-D SWE measurements in the study with healthy dogs (right: 1.51 m/s; left: 1.42 m/s). In the dogs, the shear-wave velocity was higher in the right than in the left liver, which contrasts with our study (12). In another study in dogs, the left liver and right liver were not significantly different (13). However, our results are consistent with those of prior research in humans. The left liver is surrounded by numerous anatomical structures, including the diaphragm, stomach, and aorta, and may be influenced by respiratory fluctuations, the presence of food in the stomach, and the pulsation of the aorta (30, 31). The inconsistency in the findings between dogs' and cats' right and left liver could be due to anatomical differences. In humans and cats, the liver is more right-sided, whereas it has a relatively even distribution in dogs (32). Therefore, the organs in the left cranial abdomen can have more effect in the measurement of 2-D SWE of the left liver in cats than in dogs. In human medicine, SWE measurements for liver fibrosis are generally made on the right side of the liver due to the variability of measurements in the left liver and better correlation of right liver measurements with the stage of liver fibrosis (9, 33). Therefore, standardized reference values should be applied to each side of the liver.

The 2-D SWE values did not differ according to age, weight, and BCS in our study. In the previous study evaluating 2-D SWE of the liver in dogs, the measurements were performed with young beagle dogs. Since there was no large variability between dogs, a comparison of the values by age and weight was not performed (12, 13). However, the results in our study on the effect of age, weight, and BCS are consistent with the majority of previous studies in human medicine, which revealed a lack of influence on shear wave values by age, weight, and body conformation (9, 34, 35). The depth of the measurements also had no significant effect on liver stiffness of cats in our study. In phantom studies using 2-D SWE, tissue stiffness was significantly different between measurement depths (3 vs. 6 cm). This was considered to be due to the progressive attenuation of the acoustic push pulse while crossing the tissue. However, that study only used a curved transducer, not a linear transducer (36). In another study, the curvilinear transducer had measurement bias by depth, but the linear transducer had little dependence on depth. This was considered an effect of the undesired intensity field of the push beam in the curvilinear transducer (37). In the study of 2D-SWE in livers of healthy dogs, there were two different depths from the liver capsule (5–10 mm, 10–15 mm). Shear-wave speed was higher in the far field, but the effect of the depth was questionable because there was only a significant difference in the left liver with an intercostal scan, which had a poor interobserver agreement in the far field (13). However, in a study of pSWE in the liver of healthy dogs, there was a negative significant correlation between depth and shear-wave velocity (38). We used a linear transducer; therefore, there was a low effect of the undesired intensity field of the push beam. Additionally, considering the small body size of cats compared to humans and dogs, there is less variation in measurement depth.

Failure or unreliable 2-D SWE measurement was higher in overweight cats with a BCS ≥ 6 (27.78%) than in cats with a BCS < 6 (5.88%). However, there was no significant difference in the technical failure and unreliable measurements in the statistical analysis by overweight. The insignificant statistical difference would be affected by the small number of samples. Obesity is a contributing factor to the poor reliability of the liver 2-D SWE measurement in the various reports of human medicine (39, 40). Overweight is a common condition in adult cats; in a study evaluating overweight cats (BCS ≥ 6), 45% of the included cats were overweight (41), which was similar to the 51.43% rate identified in this study. Given the high prevalence of overweight in the cat population, it is necessary to clarify whether overweight affects 2-D SWE measurements in clinical situations.

Gabapentin was prescribed if necessary, at the request of the owner, to reduce stress, decrease aggression, and increase compliance during examinations. Gabapentin has anxiolytic properties and is used in the treatment of chronic pain and epilepsy. Its use as an anxiolytic agent at an oral dose of 100 mg was well-tolerated by most cats in previous studies, with a significant reduction in stress-related behaviors (42, 43). Administration of gabapentin did not affect 2-D SWE measurement values or the rate of failure and unreliable measurements to obtain data.

This study has several limitations. First, the study was performed with a limited sample size. The possible influence of sex on neutering status and breed of the cats could not be estimated due to the small number of each group in this study. Further studies with a larger number of subjects will be required. Second, this study investigated 2-D SWE in clinically normal cats only. To evaluate the clinical applicability of 2-D SWE, this approach should be implemented in various clinical situations, such as inflammation, lipidosis, and tumor lesions, as well as in normal populations.

In conclusion, we demonstrated that 2-D SWE measurement of the liver in clinically healthy adult cats is not influenced by age, weight, and BCS and can be performed without anesthesia and breath-holding, with good reliability and high interobserver agreement. The values determined here can form the basis for reference elastography values for evaluation of the feline liver.

## Data Availability Statement

The raw data supporting the conclusions of this article will be made available by the authors, without undue reservation.

## Ethics Statement

The animal study was reviewed and approved by the Seoul National University Institutional Animal Care and Use Committee. Written informed consent was obtained from the owners for the participation of their animals in this study.

## Author Contributions

KKi, MC, and JY contributed to the conception and design of the study. KKi, JL, JS, Y-sJ, and MJ contributed to data acquisition. KKi and JL performed the data interpretation. KKi performed the statistical data analysis and the interpretation. KKi, KKa, and JY drafted the manuscript. All authors revised the manuscript and gave their final approval.

## Conflict of Interest

The authors declare that the research was conducted in the absence of any commercial or financial relationships that could be construed as a potential conflict of interest.
